# Surface Treatment of PEOT/PBT (55/45) with a Dielectric Barrier Discharge in Air, Helium, Argon and Nitrogen at Medium Pressure

**DOI:** 10.3390/ma11030391

**Published:** 2018-03-07

**Authors:** Pieter Cools, Mahtab Asadian, Wannes Nicolaus, Heidi Declercq, Rino Morent, Nathalie De Geyter

**Affiliations:** 1 Department of Applied Physics, Research Unit Plasma Technology (RUPT), Faculty of Engineering and Architecture, Ghent University, Sint-Pietersnieuwstraat 41, B4, 9000 Ghent, Belgium; mahtab.asadian@ugent.be (M.A.); wannes.nicolaus@ugent.be (W.N.); rino.morent@ugent.be (R.M.); 2 Department of Basic Medical Sciences, Tissue Engineering Group, Faculty of Medicine and Health Sciences, Ghent University, De Pintelaan 185, B3, 9000 Ghent, Belgium; heidi.declercq@ugent.be

**Keywords:** non-thermal plasma technology, PEOT/PBT, plasma activation, surface analysis, human foreskin fibroblasts, medium pressure, dielectric barrier discharge

## Abstract

This work describes the surface modification of 300PEO-PEOT/PBT 55/45 thin films using a medium pressure dielectric barrier discharge system operated in argon, helium, nitrogen or dry air to improve cell-surface interactions of this established biomaterial. The first part of the paper describes the optimization of the plasma processing parameters using water contact angle goniometry. The optimized samples are then characterized for changes in surface topography and surface chemical composition using atomic force microscopy (AFM) and X-ray fluorescence spectroscopy (XPS) respectively. For all plasma treatments, a pronounced increase in surface wettability was observed, of which the extent is dependent on the used plasma discharge gas. Except for dry air, only minor changes in surface topography were noted, while XPS confirmed that the changes in wettability were mainly chemical in nature with the incorporation of 5–10% of extra oxygen as a variety of polar groups. Similarly, for the nitrogen plasma, 3.8% of nitrogen polar groups were additionally incorporated. Human foreskin fibroblast (HFF) in vitro analysis showed that within the first 24 h after cell seeding, the effects on cell-surface interactivity were highly dependent on the used discharge gas, nitrogen plasma treatment being the most efficient. Differences between untreated and plasma-treated samples were less pronounced compared to other biodegradable materials, but a positive influence on cell adhesion and proliferation was still observed.

## 1. Introduction

Biodegradable thermoplastics such as polylactic acid (PLA), polycaprolactone (PCL), polyglycolic acid (PGA), polylactic-glycolic acid (PLGA) and poly (3-hydroxybutyrate-co-3-hydroxyvalerate) (PHBV) are widely used as structural materials in tissue engineering due to their interesting material characteristics such as a high strength to weight ratio, thermal stability, reproducibility, ease of acquisition, biocompatibility and controlled degradation rates [1,2,3,4]. As a result, research groups focusing on biomedical applications have been using these materials as such, or exploited them for the fabrication of 3D scaffolds and nanofiber mats [5]. Their applications range from support structures for musculoskeletal tissue regeneration over bone fillers and nerve guides, to dermal implants [6,7]. Although biocompatible, the bioactivity of the materials is usually limited, preventing effective cell adhesion, proliferation and migration. To overcome said limitations, an extensive amount of research has been dedicated to altering the surface properties of biodegradable thermoplastics, while maintaining the useful bulk characteristics [8,9,10,11,12,13,14]. 

One of the more widely employed surface modification techniques is non-thermal plasma technology, as it is non-invasive, solvent-free as well as time and energy efficient [15,16,17,18]. In recent years, many studies have been published describing the successful modification of biodegradable polyesters (PCL, PLA, PLGA, PGA, PHBV) using non-thermal plasma treatments at medium and atmospheric pressure [9,19,20,21,22,23,24,25,26]. However, the plasma treatment of biodegradable terephthalates remains largely unexplored. 

Polyethylene oxide (300 Da)—polyethylene-oxide terephthalate/polybutylene terephthalate (300PEO-PEOT/PBT 55/45) (Polyactive™) is a biodegradable block copolymer belonging to the terephthalate ester/ether family. It is characterized by good physical properties (strength, elasticity and toughness) combined with easy processing capabilities. These interesting material properties originate from its phase separating morphology: the soft hydrophilic PEO fragments are physically cross-linked by the presence of the harder, semi-crystalline PBT fragments. Unlike materials that are chemically cross-linked, the network can be reversibly disrupted when bringing it above glass transition temperature and vice versa, making it straightforward to process [27,28,29]. Its biocompatibility has already been thoroughly studied both in vitro and in vivo [30,31,32,33]. When incubated in aqueous media, PEOT/PBT degrades via hydrolysis and oxidation, of which the rate depends on the amount of PBT present. Currently, the material has reached the clinical stage, where it has found applications as dermal implants and bone fillers [31,34,35,36]. 

Compared to biodegradable polyesters, PEOT/PBT is characterized by a more moderate hydrophobicity, thanks to the presence of the hydrophilic PEO segments in the polymer backbone [37,38]. Still, compared to tissue culture plates (TCPs), a reduced cell viability and cell adhesion efficiency is observed [39]. In this study, we will therefore focus on the use of a non-thermal plasma system to enhance cell-surface interactions of PEOT/PBT. As this biopolymer consists of three different types of building blocks, each with different properties, differences in plasma treatment efficiency and cell-surface interactions are to be expected compared to other biodegradable materials. Furthermore, since PEOT/PBT is characterized by a higher wettability compared to the more commonly used biodegradable polyesters, it is expected that the effects of performed plasma treatments may be less substantial. It was selected to work with a parallel-plate dielectric barrier discharge (DBD) system operated at medium pressure, as this functions for relatively large plasma volumes that exhibit a homogeneous charge distribution, without the need for extensive vacuum equipment [40]. Spin-coated PEOT/PBT films will be treated in four different atmospheres (Ar, N_2_, He and dry air) and the optimized treatment conditions will be identified via water contact angle goniometry (WCA). Changes in surface properties of the optimally treated samples will be characterized via X-ray photoelectron spectroscopy (XPS) and atomic force microscopy (AFM). Finally, the samples will be subjected to an in vitro study using human foreskin fibroblasts (HFFs) to track possible changes in cell adhesion and proliferation behavior, and to see whether cell viability can be improved. 

## 2. Materials and Methods 

### 2.1. Materials

The 300-PEO-PEOT/PBT 55/45 was acquired from PolyVation™ (Groningen, The Netherlands) in the form of pellets and processed without prior purification. Chloroform 99%, anhydrous (CHCl_3_) was purchased from Sigma Aldrich (St. Louis, MO, USA). Glass cover slips (Ø = 12 and 25 mm) were ordered from VWR (Radnor, PA, USA) and cleaned with ethanol prior to use. Ar, He, N_2_ and dry air (Alphagaz 1) were ordered from Air Liquide (Paris, France). 

Human foreskin fibroblasts (HFF-1) were acquired from ATCC (Manassas, VA, USA). DMEM glutamax, penicillin (10 U/mL), streptomycin (10 mg/mL), fetal bovine serum (FBS) and sodium pyruvate were ordered from Gibco Invitrogen (Waltham, MA, USA). Calcein AM (1 mg/mL) was purchased from Anaspec (Fremont, CA, USA) and propidium iodine was ordered from Sigma Aldrich (St. Louis, MO, USA). Yellow tetrazolium dye 3-(4,5-dimethyldiazol-2-yl)-2,5-diphenyltetrazolium bromide (MTT) was acquired from Merck Promega (Darmstadt, Germany).

### 2.2. Spin Coating of PEOT/PBT Thin Films 

PEOT/PBT pellets were mixed with CHCl_3_ (2% *w*/*w*) under constant stirring (300 rpm) until the pellets were completely dissolved. The solution was then loaded into an automatic dispenser (OPUS10, Hirschman, Heilbronn, Germany) and a fixed volume (300 µL or 150 µL) of the polymer solution was deposited on glass plates with diameters of 25 and 12 mm respectively. The spin coating itself was done using an SPS machine (SPIN 150i, Wuppertal, Germany) at a rotation speed of 2000 rpm and an acceleration speed of 400 rpm for 30 s. Samples were then stored in the dark overnight to allow for any residual solvent to evaporate. 

### 2.3. DBD System Description and Plasma Treatment Procedure

The medium pressure parallel-plate DBD reactor used in this study has already been extensively described in previous works, including a complete electrical characterization [41]. In short, a discharge is generated between 2 copper electrodes (Ø = 45 mm) covered with glass plates, acting as barrier layers. The upper electrode is connected to a 50 kHz AC high voltage source, while the lower electrode is connected to ground via a 10 nF capacitor. Samples are fixed at the center of the lower electrode, after which the system is pumped down to less than 0.05 kPa using a rotary vane pump. Subsequently, the system is flushed at 3 standard liters per minute (slm) at 50 kPa for 3 min. After purging, the gas flow is set at 1 slm and the pressure is reduced to 5 kPa after which the plasma is ignited for a predefined time, while applying a fixed discharge power. The discharge power is calculated from a voltage–charge plot, which is recorded via a digital oscilloscope (Picoscope 3204A, Pico Technology, St Neots, UK), as previously described in more detail in an earlier work [42]. To enable an objective comparison between the different plasma discharges, the results will be presented as a function of energy density (J/cm^2^). This value is calculated by multiplying the plasma exposure time with the plasma power divided by the area of the electrodes and can also be found in Table 1. 

### 2.4. Contact Angle Goniometry

Within 5 min after plasma treatment, samples were subjected to a static water contact angle analysis using a Krüss Easy Drop optical system (Krüss GmbH, Hamburg, Germany). Two microliter drops were used and the contact angles were automatically calculated within 3 s using the Laplace–Young curve fitting procedure. Each contact angle value reported in this study is the average of 7 measurements randomly distributed over a single sample with standard deviations typically varying between 0.1° and 2.5°. 

### 2.5. Atomic Force Microscopy

Possible changes in surface topography were quantified using an XE-70 AFM system (Park Systems™, Suwon, South Korea). Micrographs measuring 15 × 15 µm^2^ were recorded in non-contact mode using a silicon-based cantilever (Nanosensors™ PPP-NCHR, Neuchâtel, Switzerland). Micrographs were analyzed using the included XEP processing software (V1.8.0) and were subjected to an X–Y plane autofit procedure prior to roughness determination. For each condition, 2 different samples were analyzed at 3 random locations per sample.

### 2.6. X-ray Photoelectron Spectroscopy

XPS surface analysis was performed on a PHI 5000 Versaprobe II system (ULVAC-Physical Electronics, Chigasaki, Japan) equipped with a monochromatic Al K_α_ X-ray source (hν = 1486.6 eV) operated at 23.3 W. The vacuum in the main chamber was kept below 1 × 10^−6^ Pa during measurements. Both the survey scans and the high-resolution spectra (C1s, O1s and N1s) were recorded using pass energies of 187.85 eV (eV step = 0.8 eV) and 23.5 eV (eV step = 0.1 eV) respectively, with the hemispherical analyzer set at 45° of the sample normal. The atomic elemental composition was calculated from the survey spectra using Multipak (v 9.6.1) software. A spectrum calibration (C-C = 285.0 eV) was done prior to analysis and an iterated Shirley background was applied to determine the elemental composition using the relative sensitivity factors supplied by the manufacturer. The curve peak fitting of the individual high-resolution peaks was also done making use of Multipak after applying a Savitzky–Golay smoothing procedure. The peaks were de-convoluted using Gaussian–Lorentzian peak shapes, keeping the FWHM below 1.4 eV and the χ^2^ below 2. For each condition, 1 sample was analyzed at 4 randomly distributed locations on the sample.

### 2.7. In Vitro Analysis

The induced changes in cell-surface interactions of the plasma-treated PEOT/PBT films were examined in detail. The HFF-1 cells were cultured in DMEM glutamax medium supplemented with 15% FBS, 0.5% P/S (10 U/mL penicillin, 10 mg/mL streptomycin) and 100 mM sodium pyruvate. The cells were cultured at 37 °C in a humidified atmosphere containing 5% CO_2_. Afterwards, the cells were seeded on the thin films (Ø = 12 mm) at a density of 40,000 cells/mL medium in 24-well suspension culture dishes and evaluated after 1 and 7 days. Cell attachment and distribution were visualized using a standard live/dead staining (Calcein AM/propidium iodide) procedure. After careful rinsing, the supernatant was replaced by 1 mL phosphate buffered saline (PBS) solution supplemented with 2 µL (1 mg/mL) calcein AM and 2 µL (1 mg/mL) propidium iodide. The cultures were incubated for 10 min at room temperature, washed with PBS solution and subsequently evaluated by fluorescence microscopy (Type U-RFL-T, Olympus, Tokyo, Japan, XCellence Pro software v1.2). The cell count analysis was done using the particle count module of ImageJ (v1.48) and was repeated for 3 samples per condition. 

A colorimetric MTT assay, using the yellow tetrazolium dye 3-(4,5-dimethyldiazol-2-yl)-2,5-diphenyltetrazolium bromide was also performed to quantify cell viability via colorimetry. One and 7 days after cell seeding, the culture medium was replaced by 0.5 mL (0.5 mg/mL) MTT reagent. Subsequently, the samples were incubated at 37 °C in a humidified atmosphere containing 5% CO_2_ for 4 h, after which the samples were removed from the MTT reagent and placed in a lysis buffer (1% Triton-X100 in isopropanol/0.04 M HCl) at 37 °C for 30 min to solubilize the water-insoluble formazan. Afterwards, 200 µL of the formazan solution was transferred to a 96-well plate and the absorbance of the colored solution at 580 nm was measured using a spectrophotometer (Universal microplate reader EL 800, BioTek Instruments, Winooski, VT, USA). All results were normalized to the cell metabolic activity measured on day 7 for the tissue culture plates (TCPs) and are the average of 8 measurements.

## 3. Results and Discussion

### 3.1. Analysis of the Wettability

**Figure 1:**
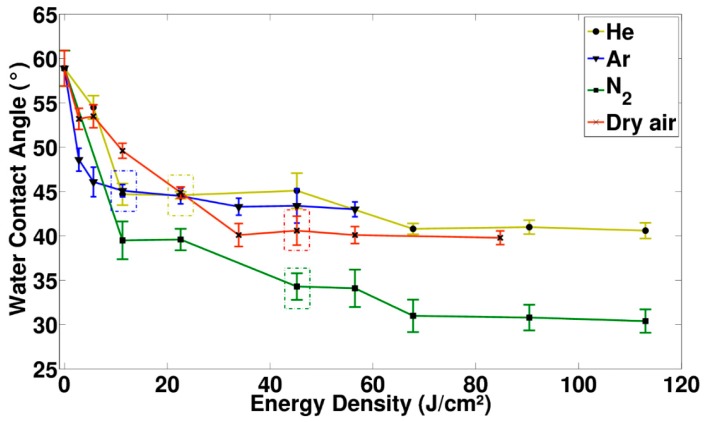
Results of water contact angle goniometry as a function of energy density. The optimized conditions used for further experiments have been marked with a box in their respective color.

Figure 1 gives an overview of the observed changes in wettability as a function of energy density, when exposing the PEOT/PBT spin-coated thin films to the four selected discharge gases (Ar, He, N_2_ and dry air). For the untreated thin films, the water contact angle (WCA) was found to be approximately 59°, which is slightly higher than the values typically found in other scientific literature [43]. When increasing the energy density by increasing the plasma treatment time, a progressive decrease in WCA was detected until a steady-state region was reached, similar to what had been observed for the plasma treatment of other thermoplastics [18,44,45,46,47]. As prolonged treatments had no further influence on the surface wettability, the start of the steady-state region was considered to be the optimized treatment for each discharge gas and the corresponding energy density and WCA values have been marked by a box in Figure 1. Depending on the discharge gas, the steady-state WCA was situated between 45° (Ar) and 31° (N_2_). Said results imply that the induced changes in surface topography and chemical composition differed between discharge gases, which is in close agreement to what has been found in scientific literature for other biodegradable polymers [48]. In general, it is well-known that thermoplastics that are plasma treated will suffer from hydrophobic recovery over time, the amount depending on its direct environment and crystalline character of the polymer. Studies, both within our group as well as by other groups, have shown that such hydrophobic recovery can be minimized when storing the samples under proper conditions (vacuum, low temperatures). As the polymer in this work will be primarily used in biomedical applications, it can simply be stored in the cell-medium prior to its use, as several studies have shown that storage of a plasma-treated polymer in a polar medium, largely preserves the induced changes in surface wettability [49,50,51,52,53,54]. To better understand the observed differences in the WCA evolution between the discharge gases, the surface topography (AFM) and the surface chemical composition (XPS) will be studied in more detail in the following sections, using the optimized treatment conditions as determined by WCA (Ar: 11.3 J/cm^2^; He: 22.6 J/cm^2^; N_2_:45.2 J/cm^2^ and Dry air: 45.2 J/cm^2^).

### 3.2. Changes in Surface Topography 

**Figure 2:**
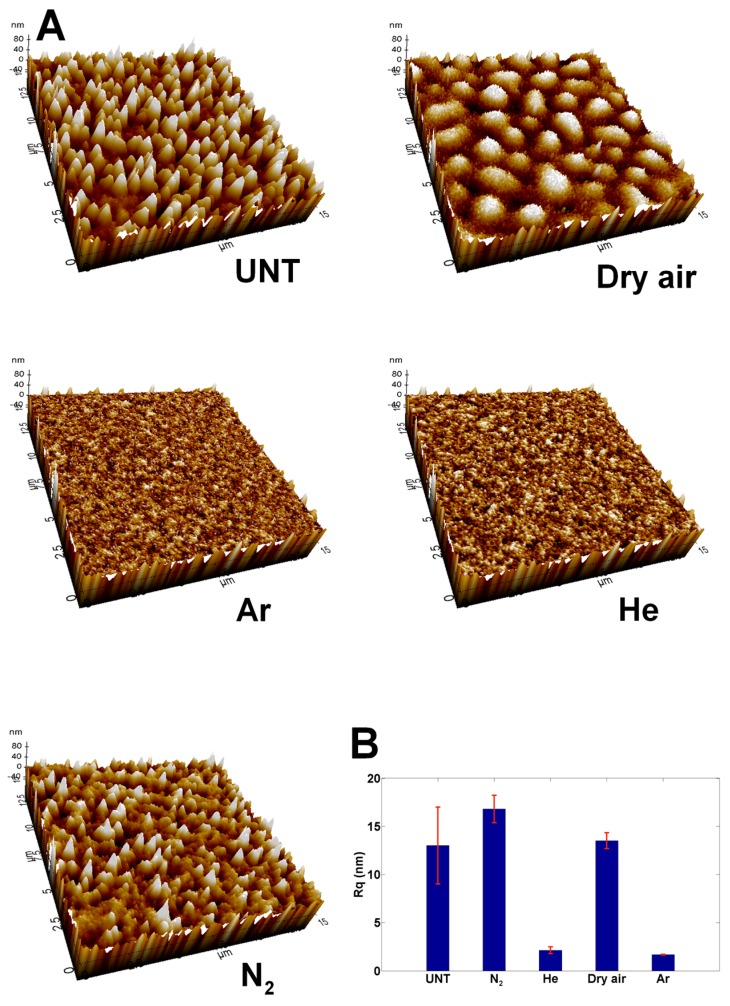
(**A**) 15 × 15 µm^2^ atomic force microscopy (AFM) micrographs before and after different optimized plasma treatments (Ar: 11.3 J/cm^2^; He: 22.6 J/cm^2^; N2: 45.2 J/cm^2^ and Dry air: 45.2 J/cm^2^). Z scale is set at 100 nm for all samples; (**B**) overview of the Rq roughness changes after the different optimized plasma treatments.

The AFM images of PEOT/PBT spin-coated films were recorded before and after each optimized plasma treatment to examine the effects that different discharges had the surface topography and roughness (see Figure 2). Although relatively subtle, some differences were observed when comparing the micrographs before and after exposure to He and Ar plasma: the most pronounced peaks present on the untreated sample (UNT) had been etched away by these plasma treatments and a more uniform roughness distribution could be observed. These alterations in surface topography were also reflected by the obtained changes in root mean square roughness values (R_q_), where a substantial decrease in surface roughness was observed. When linking this to the obtained WCA results, these results feel counterintuitive, as a decrease in surface roughness is usually linked to an increase in the WCA for hydrophilic surfaces [55]. Thus, it can be concluded that the observed decreases in the WCA after He and Ar plasma treatments were predominantly caused by changes in surface chemical composition, which was later further validated. For the N_2_ plasma treatment, no significant changes were observed in either surface topography or surface roughness values, whereas the biggest change in wettability was found for N_2_ plasma-treated surfaces. Hence, that modification must also have been primarily chemical in nature. For the air plasma treatment, a completely different surface topography was observed after the plasma treatment with the formation of hemispherical protrusions on the surface. As a result, the surface roughness value of this sample was remarkably higher compared to the Ar and He plasma treatments and was only slightly lower than the roughness recorded for the N_2_-treated/untreated PEOT/PBT films. The main reason for this difference in etching behavior could be linked to the difference in the active species present in the plasma discharge, as significantly more oxygen molecules were present in the air discharge. The presence of oxygen molecules in the plasma would have generated atomic oxygen species, which are known to be excellent for etching [56]. As PEOT/PBT is a copolymer consisting of harder and softer fragments, it was to be expected that during most plasma treatments the softer parts would have been etched preferentially, resulting in the observed surface topography, the extent of which was dependent on the type of discharge [57,58]. The differences in surface topography between the air plasma treatment and the other plasma treatments form part of the explanation as to why each treatment resulted in a different treatment efficiency. The other aspect, the surface chemical composition, will be discussed below and will give more insights as to why the WCA values were so different between the Ar, He and N_2_ treatments, although their surface topography was comparable.

### 3.3. Changes in Surface Chemical Composition

**Figure 3:**
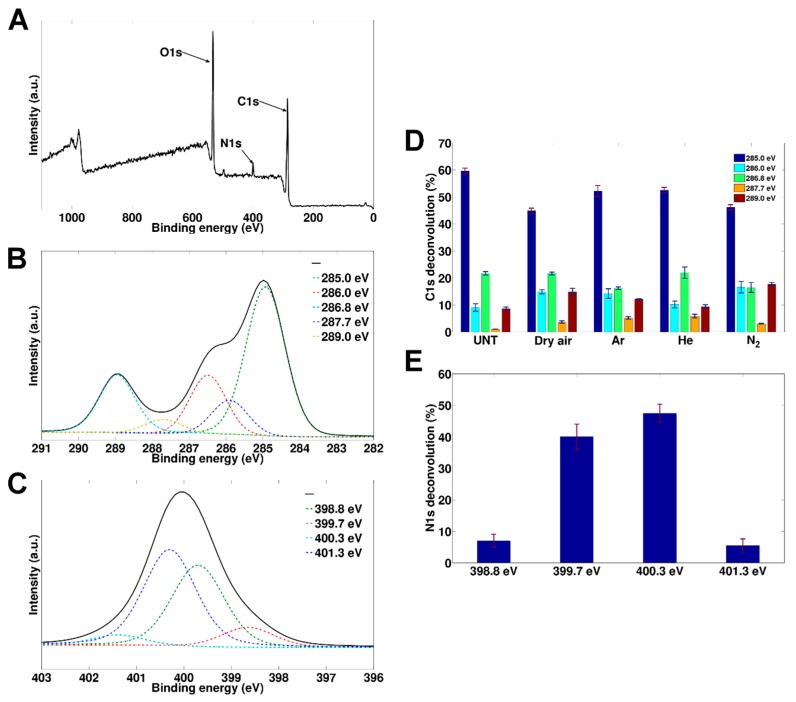
(**A**) Survey spectrum of N_2_ plasma-treated PEOT/PBT; (**B**) example of a C1s deconvolution (N_2_ plasma-treated sample); (**C**) example of an N1s deconvolution (N_2_ plasma-treated sample); (**D**) C1s peak deconvolution results for the different treatments; (**E**) N1s deconvolution results of the N_2_ plasma-treated surface.

To evaluate the types of functional groups introduced during the various plasma treatments, all optimized samples were subjected to a detailed XPS analysis, the results of which are gathered in Figure 3 and Table 2. The elemental composition of the untreated PEOT/PBT films is in close accordance to what is theoretically expected, within the margin of error: 76.4 ± 0.4% C and 23.6% ± 0.4% O vs. the theoretical 74.9% C and 25.1% O (Table 2). After plasma treatment, an increase in oxygen was observed for all discharge gases, ranging from 28.6 ± 0.1% (He) to 33.2 ± 0.2% (dry air) with Ar and N_2_ plasma-treated samples possessing intermediate oxygen amounts (approximately 30.5 ± 0.6% and 30.9 ± 1.6%) (Table 2). In addition to the incorporation of O-rich functional groups, the N_2_ plasma-treated films also contained a small percentage of nitrogen (3.7 ± 0.5%) suggesting also the incorporation of N-rich functional groups (Table 2). For the plasma treatment in a dry air atmosphere, the incorporation of oxygen directly occurred during plasma treatment as O_2_ was present in the medium. For the other treatments, the incorporation of oxygen could have directly occurred due to the presence of residual air and moisture in the plasma reactor. However, it was anticipated that oxygen incorporation mostly took place via secondary reaction pathways as the plasma-induced radical sites and meta-stables on the surface would react with oxygen molecules present in ambient air once the samples were taken out of the plasma reactor [59,60]. 

To better understand what types of polar functional groups were introduced by the different discharge gases, detailed C1s peaks were de-convoluted, an example of which is shown in Figure 3B. For the untreated PEOT/PBT, peaks at 285.0 eV (C-C/C-H), 286.0 eV (C-C=O), 286.8 eV (C-O or C-O/C-N for N_2_ plasma-treated samples) and 289.0 eV (O-C=O or O-C=O/N-C=O for N_2_ plasma-treated samples) could be identified at ratios that were close to what was expected based on the molecular structure of the polymer. Based on the C1s fitting, quantitative information could be obtained for each of the aforementioned chemical bonds, the results of which are gathered in Figure 3D. After the plasma treatments, one additional peak at 287.7 eV (C=O) was distinguished, while the relative concentrations of the other peaks also substantially changed. The relative concentration of the peak at 285.0 eV decreased for all samples, which made sense based on the results obtained from the XPS surveys which indicated an addition of oxygen/nitrogen atoms upon plasma treatment. The peak at 286.8 eV (C-O/C-N) seemed to be unchanged (He, Dry air) or even slightly decreased (Ar, N_2_), which was in sharp contrast to what was observed for most other polymers in scientific literature, where usually a pronounced increase of the C-O/C-N bonds was observed [44,61,62,63]. Most likely this unusual behavior could be linked to the preferential plasma etching of the softer PEO regions in the polymer, resulting in a depletion of the C-O bonds present in the backbone of the polymer. This decrease in the C-O groups was almost fully compensated by the introduction of new C-O functional groups in the cases of dry air and helium plasma treatment, as is evident in Figure 3D. In the cases of Ar and N_2_ plasma treatment, the additional incorporation of the C-O groups (and the C-N groups in the case of N_2_ plasma treatment) during the plasma treatment was, however, only able to partly compensate for this loss in the C-O groups, resulting in a decreasing trend in the relative concentration of the peak at 286.8 eV. For all plasma treatments, there was also the introduction of a new peak at 287.7 eV, corresponding to aldehyde/ketone functional groups, a peak which was not present in the native polymer and which was also typically observed for plasma treatments of other biodegradable polyesters [44,61,62,63]. This C=O group incorporation was slightly more pronounced on the Ar and He plasma-treated PEOT/PBT films. For the O-C=O/N-C=O peak at 289.0 eV, notable discrepancies could also be noted between the different plasma treatments. All discharge gases were responsible for an increase of said peak compared to the untreated one, but whereas for the He treatment only an additional 1% was noted, which could still be considered within the margin of error, the Ar, dry air and N_2_ treatments were responsible for an increase in the relative concentration of O-C=O/N-C=O groups with 4%, 6% and 9% respectively. As these were the largest observed changes in relative concentration, it was expected that the corresponding chemical groups resulted in the most pronounced impact on the changes in wettability. Linking the overall results back to the changes in wettability, a close correlation could be found between the lowest attainable WCA values and the lump sum of the introduced polar functional groups: the higher the O(+N) incorporation efficiency, the lower the WCA value became. These results thus clearly indicated that the observed differences in the optimized WCA values were mainly chemical in nature.

As mentioned earlier, the exposure of the PEOT/PBT to the N_2_ discharge resulted in both oxygen incorporation, and also in the incorporation of a substantial amount of nitrogen-containing functional groups. To distinguish the C-N and the O=C-N within the C1s peak envelope is rather difficult due to substantial overlap with the C-O and the O-C=O peaks respectively and was therefore not attempted in this study. However, to have an idea of the relative amounts of C-N and O=C-N that were introduced during the treatment, the N1s peak had therefore also been de-convoluted and the result is shown in Figure 3C.

The averaged quantitative results are given in Figure 3E and the following trends were observed: small amounts of primary amines (398.8 eV) and charged amines (401.3 eV) were found (around 5%), but most of the incorporated nitrogen groups were either secondary amines (399.7 eV) or amides (400.3 eV) (40% and 48% respectively). Neither type of nitrogen-functional group was well-known to stimulate cell-surface interactions, something which would have needed to be confirmed by in vitro analysis [64], which was performed and is detailed in the following section. 

### 3.4. Improvement of Cell-Surface Interactions

**Figure 4:**
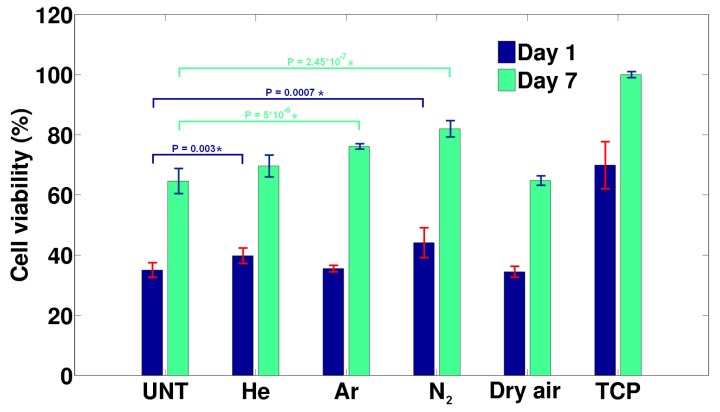
Human foreskin fibroblast (HFF) cell viability 1 and 7 days after seeding for the different treatments (Ar: 11.3 J/cm^2^; He: 22.6 J/cm^2^; N_2_: 45.2 J/cm^2^ and Dry air: 45.2 J/cm^2^) relative to tissue culture plates (TCPs). Samples marked with an * are significantly different with a 99% certainty.

Figure 4 gives an overview of the HFF cell viability 1 and 7 days after cell seeding. After the first day, the differences between untreated PEOT/PBT and the plasma-treated samples were relatively small with only He and surprisingly N_2_ plasma treatments which resulted in a significant improvement in cell adhesion. Still, with only a 60% viability compared to TCP, the results could only be considered mediocre. One week after seeding, the effects of the performed plasma treatments on cell viability were slightly more pronounced, but in this case the Ar and again the N_2_ treatments were found to have induced a significant improvement in cell proliferation, whereas dry air and He plasma treatments were found to have only a minor non-significant effect. For the N_2_ plasma-activated surfaces, the cell viability was found to be only 20% less compared to the TCP, indicating a relatively good cell-surface compatibility. Additionally, as the cell viability had increased by almost 20% compared to the untreated PEOT/PBT, it could also be concluded that the nitrogen plasma had a pronounced positive effect on the bioactivity of the treated surface. However, it needs to be mentioned that compared to non-thermal plasma treatments conducted on other biodegradable polymers, the induced effects on cell-surface interactions seemed to be much more modest for PEOT/PBT [19,20,65,66]. The explanation of this is two-fold: first, the wettability of the pristine PEOT/PBT co-polymer was considerably higher, which resulted in a much-improved initial cell viability of the untreated material, which made the possible impact of the treatment less pronounced. Secondly, as some of the plasma discharges preferentially etched away the softer, hydrophilic PEO segment of the polymer, it was to be expected that part of the initial cell-interactivity would be lost and would need to be entirely replaced by plasma-activated groups, thereby reducing the overall positive plasma effect.

**Figure 5:**
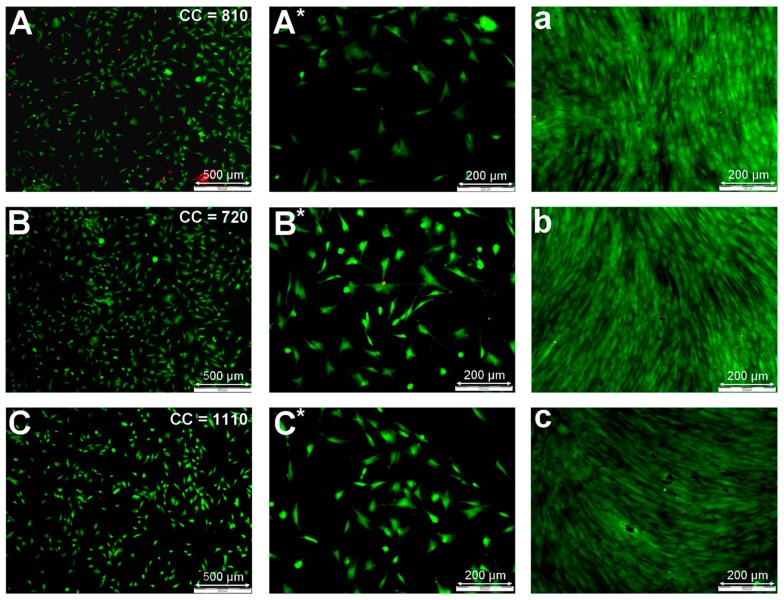

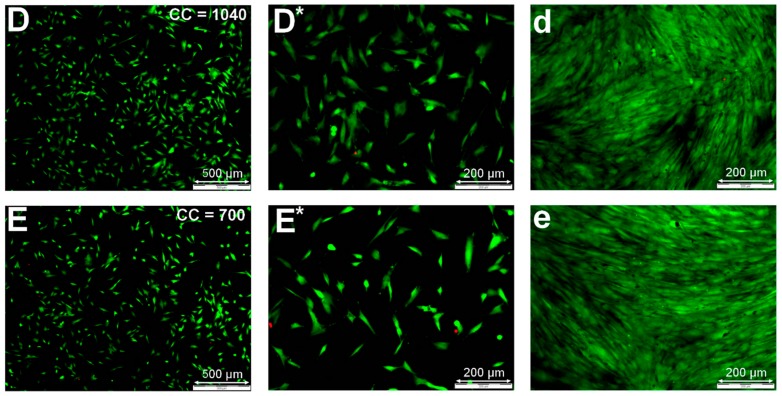
Life/dead fluorescent microscopic images for the different optimized plasma treatments 1 (**A**–**E**/A*–E*) and 7 (**a**–**e**) days after HFF cell seeding. **A**/A*/**a** = untreated samples; **B**/B*/**b** = He plasma-treated (22.6 J/cm^2^); **C**/C*/**c** = Ar plasma-treated (11.3 J/cm^2^); **D**/D*/**d** = N_2_ plasma-treated (45.2 J/cm^2^); **E**/E*/**e** = Air plasma-treated (45.2 J/cm^2^). Cell count (CC) data given in 5**A**–**E** were calculated from five independent images and averaged. Standard deviations were less than 10% for all conditions.

Analysis of the life/dead fluorescent micrographs showed the cellular adhesion of the HFFs 24 h after seeding as mediocre for the untreated sample, which was in agreement with the results shown in Figure 4. The number of adhered cells was not very high; however, the adhered cells showed a nice spread-out morphology (Figure 5A). The microscopic images of the plasma-treated samples confirmed slight improvements in cell adhesion 1 day after cell seeding for He and N_2_ plasma-treated surfaces, as shown by the cell count results mentioned in Figure 5A–E, which is in agreement with the MTT results shown in Figure 4. A similar cell morphology was observed on the plasma-treated samples compared to those adhering to the untreated material. Seven days after cell seeding (Figure 5a–e), the surfaces of all samples, including the untreated one were entirely covered by a dense layer of HFF cells, showing their characteristic cell spindle shape and supra-cellular parallel orientation often observed for maturing fibroblast cultures. This again confirmed that the initial bioactivity of PEOT/PBT was already relatively high, which made it difficult to observe subtle changes in cell proliferation between the different samples, as could be obtained with the MTT analysis. 

Based on the MTT and fluorescent micrographs, it became evident that from the four different plasma treatments, the N_2_ plasma treatment had the most pronounced positive effect on cell-surface interactions. When linking back to the surface analysis results, it may be that the higher wettability was responsible for this, although, based on the differences between the optimized WCA values of the other treatments, it seemed that this alternative behavior might rather have originated from the type of incorporated polar groups (nitrogen vs. oxygen). As such, it can be concluded that N_2_ plasma treatment shows potential for the surface activation of PEOT/PBT 3D porous structures designed for tissue regeneration applications, as the cell-surface interactivity of such structures is known to be much more sensitive to even small changes in surface wettability.

## 4. Conclusions

This paper describes the effect of several plasma treatments (argon, helium, nitrogen and dry air) on PEOT/PBT thin films. Based on the results obtained from the WCA and the XPS analysis, it can be concluded that all discharge atmospheres have a pronounced positive influence on the wettability of PEOT/PBT thin films, but depending on the type of discharge atmosphere, the effects became more/less pronounced. The XPS C1s deconvolutions showed that different treatments were responsible for introducing varying degrees of incorporation of oxygen polar groups. The changes in peak intensity at 289.0 eV, correlating with the incorporation of the O=C-O groups, were strongest and showed the most variability between treatments. For the N_2_ plasma discharge, considerable amounts of nitrogen functional groups were also incorporated mainly as secondary amines and amides. AFM images showed a reduction in surface roughness for He and Ar treatments, which could be attributed to selective etching of the soft polymer regions while N_2_ treatments did not seem to have a pronounced impact on the topography. Air plasma treatments resulted in a complete change of surface topography due to more pronounced etching. Overall, it can be concluded that the induced changes in wettability were mainly the result of differences in chemical composition and not of surface roughness changes. The in vitro cell studies strongly indicated that in the first 24 h after cell seeding, the effects of the different plasma treatments were relatively modest with only the He- and N_2_-treated surfaces inducing a significant increase in cell adhesion efficiency. In contrast, 7 days after cell seeding, Ar- and N_2_-treated surfaces were found to have the most positive effect on cell proliferation. The combination of both results indicates that the N_2_ plasma treatment is, of the four used discharge gases, best suited for the enhancement of PEOT/PBT surface properties and has potential for the surface enhancement of 3D porous PEOT/PBT structures used for tissue engineering. 

## Figures and Tables

**Table 1:** Plasma treatment parameters for the different discharge gases.

Discharge Gas	Discharge Power	Treatment Time	Energy Density
Argon	3 W	0–240 s	0–56.5 J/cm^2^
Helium	6 W	0–300 s	0–113.0 J/cm^2^
Nitrogen	6 W	0–300 s	0–113.0 J/cm^2^
Dry air	3 W	0–300 s	0–84.8 J/cm^2^

**Table 2:** Overview of the surface elemental composition (in at %) as measured by XPS survey scans of the different treatments (Ar: 11.3 J/cm^2^; He: 22.6 J/cm^2^; N2: 45.2 J/cm^2^ and Dry air: 45.2 J/cm^2^).

Element	Unt	Dry air	Ar	He	N_2_
C	76.4 ± 0.4	66.8 ± 0.2	69.5 ± 0.7	71.4 ± 0.1	65.4 ± 1.5
O	23.6 ± 0.4	33.2 ± 0.2	30.5 ± 0.7	28.6 ± 0.1	30.9 ± 1.3
N	-	-	-	-	3.7 ± 0.5
